# Genomes of two type strains of the *Rhizobium tropici* group: *R. calliandrae* CCGE524^T^ and *R. mayense* CCGE526^T^

**DOI:** 10.1128/MRA.00472-23

**Published:** 2023-08-04

**Authors:** Fernanda Terezinha Moura, Luisa Caroline Ferraz Helene, Milena Serenato Klepa, Renan Augusto Ribeiro, Marco Antonio Nogueira, Mariangela Hungria

**Affiliations:** 1 Department of Biochemistry and Biotechnology, Universidade Estadual de Londrina, Londrina, Paraná, Brazil; 2 Embrapa Soja, Soil Biotechnology Laboratory, Londrina, Paraná, Brazil; 3 CAPES, SBN, Brasília, Distrito Federal, Brazil; 4 CNPq, Brasília, Distrito Federal, Brazil; The University of Arizona, Tucson, Arizona, USA

**Keywords:** *Rhizobium tropici*, *Rhizobium calliandrae*, *Rhizobium mayense*, biological nitrogen fixation, nodulation, plasmid, symbiosis

## Abstract

The genome sequences of two nitrogen-fixing type strains of the *Rhizobium tropici* group were obtained: *Rhizobium calliandrae* CCGE524^T^ and *R. mayense* CCGE526^T^. Genomic analyses confirmed their taxonomic position and identified three complete sequences of the *repABC* genes, indicative of three plasmids, one of them carrying symbiotic genes.

## ANNOUNCEMENT

The *Rhizobium tropici* group encompasses species of nitrogen-fixing rhizobia that, in general, are very promiscuous in nodulating several legumes and may contribute with high rates of nitrogen fixation (1 - 3). *Rhizobium calliandrae* CCGE524^T^ and *R. mayense* CCGE526^T^ were isolated from an effective nodule of the medicinal legume *Calliandra grandiflora* in rainforests of Chiapas, Mexico (4). Genetic characterization based on 16S rRNA and housekeeping genes (*atpD*, *recA*, and *rpoB*) positioned the two species into the *R. tropici* clade (4).

Nowadays, the use of genomic parameters is highly encouraged in studies of taxonomy and phylogeny of bacteria, including rhizobia (5). As there were no genomes available for these two type strains, we performed next-generation sequencing to obtain high-quality genomes. The type strains were recovered from cryopreserved stocks at −80°C in modified yeast extract mannitol (YM) culture medium (6) with 30% glycerol (vol/vol) from the “Diazotrophic and Plant Growth Promoting Bacteria Culture Collection of Embrapa Soja,” in Londrina, Paraná, Brazil, grown in liquid modified YM medium, and incubated at 28°C for 3 days. Total DNAs were extracted with the DNeasy Blood and Tissue kit (Qiagen). Libraries were constructed with Nextera XT kit, and paired-end sequencing was performed on the NextSeq 1000 platform (Illumina) at Embrapa Soja. The reads were assembled with the A5-MiSeq pipeline (*de novo*) v.20140604 (7), with default quality control parameters, trimming sequence adapters, and low-quality regions with Trimmomatic (8), errors were corrected with String Graph Assembler’s (SGA’s) k-mer-based algorithm (9), and annotated with the NCBI Prokaryotic Genome Annotation Pipeline (PGAP), version 6.4 (10). ANI (Average Nucleotide Identity) (11) and digital DNA-DNA hybridization (dDDH) (12, 13) were calculated with the species of the *R. tropici* clade.

The two strains present genomes of similar sizes, estimated at 7,547,379 bp for *R. calliandrae* CCGE524^T^ and 7,367,519 bp for *R. mayense* CCGE526^T^. The genome coverages ranged from 140- to 143-fold, and the G + C contents were also similar for the two strains. These and other genomic parameters are shown in Table 1.

**TABLE 1:** Statistical parameters and genomic comparisons for CCGE524^T^ and CCGE526^T^

Statistical parameters	Data of the strains			
	CCGE524^T^		CCGE526^T^	
16S accession number	JX855162		JX855172	
Genome accession number	JARFYN000000000		JARFYM000000000	
Raw reads	SRR24766110		SRR24767366	
Genome size (bp)	7,547,379		7,367,519	
G + C content (%)	59.1		59.7	
*N*_50_ (bp)	97,471		156,426	
Read length (bp)	100		100	
Number of contigs	273		154	
No. of reads in total	11,243,138		10,565,130	
No. of coding sequences	7,364		6,948	
No. of RNAs	56		56	
Coverage (×)	143		140	
**Species of the *R. tropici* clade**	*R. calliandrae* CCGE524^T^		*R. mayense* CCGE526^T^	
	ANI (%)	dDDH (%)	ANI (%)	dDDH (%)
*R. calliandrae* CCGE524^T^	100	100	91.24	45.6
*R. dioscoreae* S-93^T^	81.95	24.1	82.29	24.6
*R. freirei* PRF 81^T^	82.43	24.9	82.73	25.4
*R. hainanense* CCBAU 57015^T^	82.16	24.4	82.44	24.9
*R. jaguaris* CCGE525^T^	89.77	40.7	90.67	43.7
*R. leucaenae* CFN 299^T^	85.94	31.8	86.69	33.0
*R. lusitanum* P1-7^T^	82.70	25.5	83.06	26.0
*R. mayense* CCGE526^T^	91.24	45.6	100	100
*R. miluonense* HAMBI 2971^T^	82.15	24.6	82.60	25.2
*R. multihospitium* HAMBI 2975^T^	82.11	24.3	82.51	24.9
*R. paranaense* SEMIA 4064	86.29	32.5	87.02	33.8
*R. rhizogenes* NBRC 13257^T^	83.85	27.9	84.43	28.8
*R. tropici* CIAT 899^T^	82.47	24.7	82.59	24.9
*R. vallis* CCBAU 65647^T^	80.08	21.8	80.37	22.0

Three complete sequences of the *repABC* genes (plasmid replication genes) were detected in the two strains, that may indicate three plasmids, one of which carrying the nodulation and nitrogen fixation genes. We may suppose that the three plasmids correspond to the same organization reported for *R. jaguaris* CCGE525^T^, another close species of the *R. tropici* group that carries a chromid, a symbiotic plasmid, and an additional plasmid (14). In addition, the genomes of *R. jaguaris* and of the two type strains CCGE524^T^ and CCGE526^T^ share the same organization of the genes *nodABCD* and *nifABEHNQSTUWXZ*.

The genome comparison of the species of the *R. tropici* clade indicated ANI values of 80.08%–91.24% for *R. calliandrae* CCGE524^T^ and of 80.37%–91.24% to *R. mayense* CCGE526^T^, while the dDDH values ranged from 21.8% to 45.6%, and 22.0% to 45.6%, respectively (Table 1). Therefore, the values of ANI and dDDH are below the species delineation cutoffs established in literature, of 70% and 95%–96%, respectively (5, 15), confirming the taxonomic status of the two strains.

## Data Availability

The whole-genome shotgun project for *Rhizobium calliandrae* CCGE524^T^ has been deposited at DDBJ/EMBL/GenBank under the GenBank accession number JARFYN000000000 (accession numbers BioProject PRJNA940114, BioSample SAMN33553174). The version described in this paper is JARFYN000000000. The whole-genome shotgun project of *Rhizobium mayense* CCGE526^T^ has been deposited at DDBJ/EMBL/GenBank under the GenBank accession number JARFYM000000000 (accession number BioProject PRJNA940111, BioSample SAMN33553147). The version described in this paper is JARFYM000000000.

## AFTER EPUB

[This article was published on 4 August 2023 with missing information in the "Data availability" statement and an incorrect affiliation for Renan Augusto Ribeiro. The statement and affiliation were corrected in the current version, posted on 9 August 2023.]
